# Clinical significance of day 5 peripheral blast clearance rate in the evaluation of early treatment response and prognosis of patients with acute myeloid leukemia

**DOI:** 10.1186/s13045-015-0145-1

**Published:** 2015-05-10

**Authors:** Cong Yu, Qing-lei Kong, Yun-xiang Zhang, Xiang-qin Weng, Jing Wu, Yan Sheng, Chun-lei Jiang, Yong-mei Zhu, Qi Cao, Shu-min Xiong, Jun-min Li, Xiao-dong Xi, Sai-juan Chen, Bing Chen

**Affiliations:** State Key Laboratory of Medical Genomics, Shanghai Institute of Hematology, Rui Jin Hospital, affiliated to Shanghai Jiao Tong University (SJTU) School of Medicine, Collaborative Innovation Center of Systems Biomedicine, SJTU, Shanghai, China

**Keywords:** Peripheral blast clearance rate, Early treatment response, Prognosis, AML

## Abstract

**Background:**

Minimal residual disease detection in the bone marrow is usually performed in patients with acute myeloid leukemia undergoing one course of induction chemotherapy. To optimize the chemotherapy strategies, more practical and sensitive markers are needed to monitor the early treatment response during induction. For instance, peripheral blood (PB) blast clearance rate may be considered as such a monitoring marker.

**Methods:**

PB blasts were monitored through multiparameter flow cytometry (MFC). Absolute counts were determined before treatment (D_0_) and at specified time points of induction chemotherapy (D_3_, D_5_, D_7_, and D_9_). The cut-off value of D_5_ peripheral blast clearance rate (D5-PBCR) was defined through receiver operating characteristic (ROC) analysis. Prognostic effects were compared among different patient groups according to D5-PBCR cut-off value.

**Results:**

D5-PBCR cut-off value was determined as 99.55%. Prognostic analysis showed that patients with D5-PBCR ≥99.55% more likely achieved complete remission (94.6% vs. 56.1%, *P* < 0.001) and maintained a relapse-free status than other patients (80.56% vs. 57.14%, *P* = 0.027). Survival analysis revealed that relapse-free survival (RFS) and overall survival (OS) were longer in patients with D5-PBCR ≥99.55% than in other patients (two-year OS: 71.0% vs. 38.7%, *P* = 0.011; two-year RFS: 69.4% vs. 30.7%, *P* = 0.026). In cytogenetic-molecular intermediate-risk group, a subgroup with worse outcome could be distinguished on the basis of D5-PBCR (<99.55%; OS: *P* = 0.033, RFS: *P* = 0.086).

**Conclusions:**

An effective evaluation method of early treatment response was established by monitoring PB blasts through MFC. D5-PBCR cut-off value (99.55%) can be a reliable reference to predict treatment response and outcome in early stages of chemotherapy. The proposed marker may be used in induction regimen modification and help optimize cytogenetic-molecular prognostic risk stratification.

**Electronic supplementary material:**

The online version of this article (doi:10.1186/s13045-015-0145-1) contains supplementary material, which is available to authorized users.

## Background

Acute myeloid leukemia (AML) is a group of clinically and genetically heterogeneous diseases [1,2]. Despite treatment advancements in acute promyelocytic leukemia (M3), current treatment of AML is based on chemotherapy. Standard induction chemotherapy consists of anthracycline and cytarabine (3 + 7 regimen) can achieve the complete remission (CR) rate of approximately 75%, but outcome is uncertain because of the variability of individual genetic profile and drug sensitivity [3,4]. Intense chemotherapy or allogeneic hematopoietic cell transplantation (allo-HSCT) can benefit patients who are refractory or tend to relapse [5]. Early and easy monitoring of minimal residual disease (MRD) reflects treatment response in time and becomes an essential reference for patients with AML to optimize chemotherapy.

Multiparameter flow cytometry (MFC) has been used as a standard technique to track MRD in leukemia patients in the past decades [6]. In patients with acute lymphoblastic leukemia (ALL), the threshold of 0.01% of the bone marrow (BM) MRD is considered as the boundary of relapse predict index [7,8]. In AML, ambiguous threshold is approximately 0.1% and accuracy is approximately 10 times lower than that in ALL [9]. Moreover, BM MRD status in AML cannot be considered as an independent prognostic predictor, even though this status is considered as such in ALL [8].

Either in ALL or AML patients, the time point of BM MRD measurement is usually after CR, which may be late to determine early treatment response. Patients also hesitate to undergo frequent BM aspirations. Thus, peripheral blood (PB) blast clearance in early stage of chemotherapy has been extensively investigated. In ALL, encouraging results have been reported; for instance, the complete clearance of PB blasts within the first week of treatment may be related to CR achievement [10,11]. In patients with AML, the PB blast clearance rate (PBCR) is closely correlated with treatment response and survival, but sampling time point and cut-off value vary [12-16].

In this study, we assessed the prognostic value of PBCR during induction in a cohort of 96 newly diagnosed AML patients. An earlier, easier, and more accurate technique than current systems has been established to distinguish high-risk patients and to enable a prompt improvement of induction chemotherapy.

## Results

### Patient characteristics

From June 2011 to August 2014, 96 newly diagnosed *de novo* AML (non-M3) were included in the study. Patient characteristics are summarized in Table 1. Median age was 44.5 years (14–74). Median WBC counts and circulating blasts were 13.3 × 10^9^/L (range, 1.32 × 10^9^/L to 249.90 × 10^9^/L) and 3730.7/μL (range, 11.32/μL to 246,000/μL), respectively. The median percentage of BM blasts was 65% (14.5% to 98%).

**Table 1:** **Clinical characteristics of all patients and stratified by D5 peripheral blast clearance rate (D5-PBCR)**

**Characteristics**	**All patients (** ***n*** **= 96)** ^**a**^	**D5-PBCR ** **≥99.55% ** **(** ***n*** **= 37)**	**D5-PBCR ** **<99.55% ** **(** ***n*** **= 57)**	***P*** **value**
Age, y, median (range)	44.5 (14–74)	44 (15–67)	45 (14–74)	0.467
Gender (no.)				0.996
Male	61	24	37	
Female	35	13	20	
WBC count (×10^9^/L), median (range)	13.3 (1.32–249.90)	26.99 (3.2–120.9)	9.20 (1.32–249.90)	0.160
Hemoglobin (g/L), median (range)	85.0 (34.90–136.00)	89.5 (55.0–136.0)	82.0 (34.9–125.0)	0.051
Platelet (×10^9^/L), median (range)	38.0 (3.00–455.00)	38.5 (6.0–179.0)	36.0 (3.0–221.0)	0.975
Blasts in bone marrow (%), median (range)	65.0 (14.50–98.00)	71.0 (21.5–96)	60.1 (14.5–98.0)	0.038
Blasts in peripheral blood (/μL), median (range)	3730.7 (11.32–246,000)	4961.7 (479.9–124,900)	3730.7 (11.32–246,000)	0.035
FAB subtypes (no.)				0.229
M1	2	1	1	
M2	24	5	18	
M4	43	19	24	
M4Eo	2	2	0	
M5	19	7	11	
M6	1	1	0	
NA	5	2	3	
Cytogenetic-molecular risk group (no.)				0.045
Favorable	23	12	11	0.148
Intermediate	53	22	30	0.515
Unfavorable	20	3	16	0.019
LAIP (no.)				-
Cross-lineage antigen expression	63	29	34	
Asynchronous antigen expression	6	0	6	
Antigen dim/strong expression	13	6	6	
Antigen expression missing	8	4	3	
CR (%)	67 (69.79)	35 (94.6)	32 (56.1)	<0.001
MRD (+) (%)^b^	29 (55.77)	14 (50)	15 (62.5)	0.366
Relapse (%)	26 (32.50)	7 (19.44)	18 (42.86)	0.027

*NA* not available, *CR* achieved complete remission after one course of induction therapy, *LAIP* leukemia-associated aberrant immunophenotype, *FAB* French-American-British, *MRD* minimal residual disease. ^a^Two patients lacking in the peripheral blast absolute counts data of day 5 could not be further stratified into different groups according to D5-PBCR; ^b^MRD (+): In 67 patients achieved CR, LAIPs were detectable in 52 cases.

Leukemia-associated aberrant immunophenotypes (LAIPs) were identified in 72 (75%) patients with four main types: 63 cases with cross-lineage antigen expression, 6 cases with asynchronous antigen expression, 13 cases with antigen dim/strong expression, and 8 cases with antigen expression missing. The details of LAIP distribution were listed in Additional file 1: Table S1.

Among the 58 cases with normal or unavailable karyotype, *NRAS* mutations were found in 4 cases (7.14%), *NPM1* in 12 (21.43%), *FLT3-ITD* in 6 (10.71%), *FLT3-TKD* in 2 (3.51), *DNMT3A* in 12 (20.69%), *CEBPA* biallelic in 16 (28.07%), *MLL-PTD* in 4 (7.02%), and *MLL*-fusion gene was found in 2 (3.51%) patients. According to the cytogenetic-molecular prognostic risk classification, 23, 53, and 20 patients were classified into favorable, intermediate, and unfavorable groups, respectively.

CR was achieved in 69.79% (67/96) of patients after one course of induction, and LAIPs could be detected in 77.61% (52/67) at diagnosis. After induction, 29 patients (55.77%) remained MRD positive (≥0.1%) by MFC. Total relapse rate was 32.5% (26/80).

### Relationship between treatment response and PB blast reduction ratio

A rapid reduction in PB blasts was observed in the CR group vs. in the non-complete remission (NCR) group, with PB blast reduction ratios (PBRRs) of 1.09 ± 0.62 vs. 0.70 ± 0.53, 2.49 ± 0.92 vs. 1.70 ± 0.70, 3.41 ± 1.02 vs. 2.49 ± 0.95, and 3.92 ± 1.28 vs. 2.91 ± 1.13 on days 3 (D_3_), 5 (D_5_), 7 (D_7_), and 9 (D_9_), respectively (D_3_: *P* = 0.005, D_5_: *P* < 0.0001, D_7_: *P* < 0.0001, and D_9_: *P* < 0.0001; Table 2).

**Table 2:** **Correlation of PB blast reduction ratio (PBRR) with treatment response**

	**PB blast reduction ratio (Log10) ( ± SD)**			
	**Day 3**	**Day 5**	**Day 7**	**Day 9**
CR (*n* = 67)	1.09 ± 0.62	2.49 ± 0.92	3.41 ± 1.02	3.92 ± 1.28
NCR (*n* = 29)	0.70 ± 0.53	1.70 ± 0.70	2.49 ± 0.95	2.91 ± 1.13
*P* value	0.005	<0.0001	<0.0001	<0.0001
MRD (+) *n* = 23)	1.05 ± 0.60	2.43 ± 1.05	3.19 ± 1.11	3.62 ± 1.41
MRD (−) (*n* = 29)	1.25 ± 0.64	2.69 ± 0.78	3.19 ± 1.11	4.15 ± 1.13
*P* value	0.266	0.334	0.162	0.157
Relapse (+) (*n* = 25)	0.85 ± 0.45	2.00 ± 0.68	2.83 ± 0.78	3.15 ± 1.02
Relapse (−) (*n* = 54)	1.12 ± 0.67	2.59 ± 0.93	3.57 ± 0.98	4.16 ± 1.19
*P* value	0.066	0.005	0.001	0.0005
Early relapse^a^ (+) (*n* = 16)	0.80 ± 0.45	1.91 ± 0.80	2.76 ± 0.81	3.14 ± 1.10
Early relapse^a^ (−) (*n* = 63)	1.10 ± 0.65	2.52 ± 0.89	3.48 ± 0.97	4.02 ± 1.19
*P* value	0.094	0.017	0.008	0.009

*CR* achieved complete remission after one course of induction therapy, *NCR* not achieved complete remission, *MRD* minimal residual disease. ^a^Early relapse: hematologic relapse within 6 months.

In 52 patients who were under LAIP surveillance after CR, no significant differences in PBRRs were observed between MRD-positive and negative groups (Table 2). Significantly higher PBRRs were observed in non-relapse vs. relapse group, with 2.59 ± 0.93 vs. 2.00 ± 0.68, 3.57 ± 0.98 vs. 2.83 ± 0.78, and 4.16 ± 1.19 vs. 3.15 ± 1.02 on D_5_, D_7_, and D_9_, respectively (D_5_: *P* = 0.005, D_7_: *P* = 0.001, D_9_: *P* = 0.0005). In patients who experienced relapse within 6 months (early relapse), significant differences in PBRRs on D_5_, D_7_, and D_9_ were also observed (D_5_: 2.52 ± 0.89 vs. 1.91 ± 0.80, *P* = 0.017; D_7_: 3.48 ± 0.97 vs. 2.76 ± 0.81, *P* = 0.008; D_9_: 4.02 ± 1.19 vs. 3.14 ± 1.10, *P* = 0.009; Table 2).

### Determination of D5-PBCR cut-off value

The PBRR of D_5_ showed the highest significant difference between early CR and NCR groups. The difference in PBRRs related to the relapse status also showed that D_5_ was the first time point to appear with a definite prognostic value. Receiver operating characteristic (ROC) analysis was performed to evaluate the predictive power of PBRR on patients’ complete remission. The area under the curve (AUC) of D_5_ was 0.746, which was larger than that of D_7_ (0.736) and D_9_ (0.719),and hence showed more statistical significance. Thus, D_5_ was chosen as the time point to determine D5 peripheral blast clearance rate (PBCR) cut-off value.

**Figure 1:**
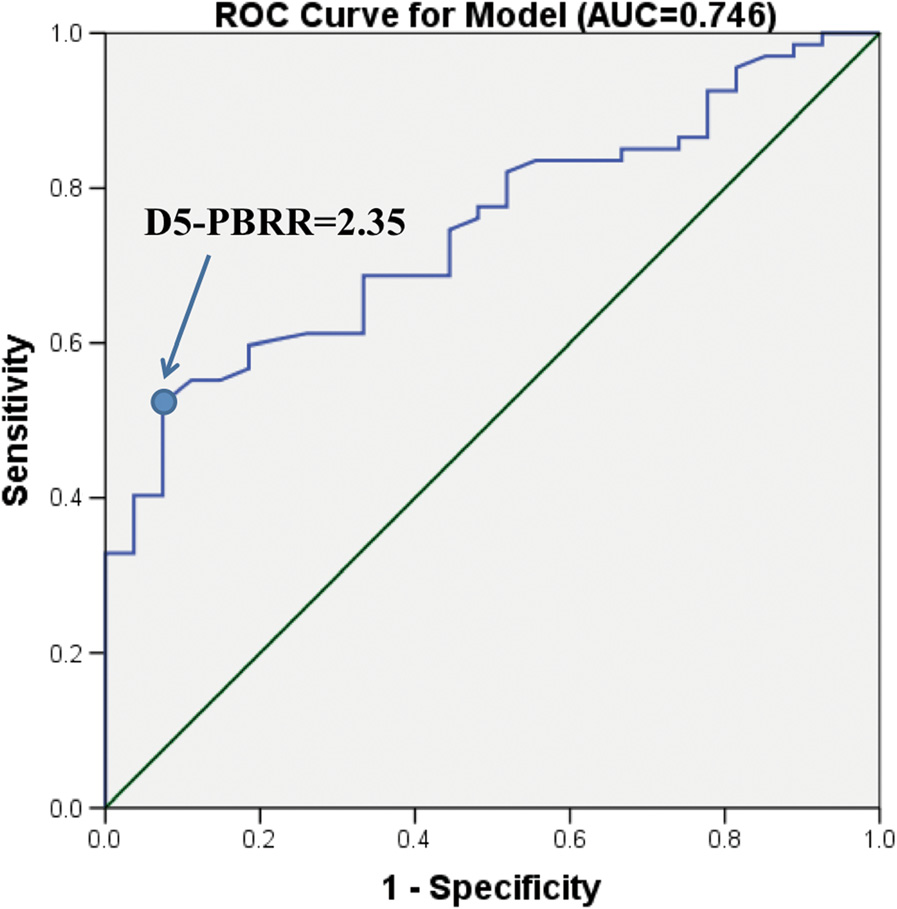
Receiver operating characteristic (ROC) curve of D5-PBRR on patients’ remission. Area under the curve (AUC) was 0.746. A cut-off value of 2.35 that maximized the sum of sensitivity and specificity of 52.2% and 92.6%, respectively, was selected.

The day 5 PB blast reduction ratio (D5-PBRR) of 2.35 was selected as the optimal cut-off, according to the maximum sum of the sensitivity and specificity with 52.2% and 92.6% on the ROC curve, respectively (Figure 1). A logarithmic value of 2.35 of PBRR is equal to the clearance rate of 99.55% initial peripheral blasts; thus, D5-PBCR was determined as 99.55%.

### Prognostic impact of D5-PBCR

As shown in the three rightmost columns of Table 1, patients were divided into two groups according to the D5-PBCR cut-off value. The two groups’ clinical characteristics, including age, gender, French-American-British (FAB) subtype, and initial WBC, were comparable. Patients with higher D5-PBCR (≥99.55%) presented higher percentage of BM blasts (71.0% vs. 60.1%, *P* = 0.038), and higher PB blast counts (4961.7/μL vs. 3730.7/μL, *P* = 0.035) at diagnosis.

Patients with higher D5-PBCR (≥99.55%) were more likely to achieve CR (94.6% vs. 56.1%, *P* < 0.001) and exhibit less relapse rate (19.44% vs. 42.86%, *P* = 0.027). However, post-induction BM MRD showed no significant difference between the two D5-PBCR groups (MRD positive: 50% vs. 62.5%, *P* = 0.366).

In molecular studies, a trend of more *NRAS* (14.8% vs. 0%, *P* = 0.051), *CEBPA* biallelic mutations (37.0% vs. 20.7%, *P* = 0.142), and less *MLL-PTD* mutations (3.7% vs. 10.3%, *P* = 0.612) were observed in the D5-PBCR ≥99.55% group (Additional file 2: Table S2).

**Figure 2:**
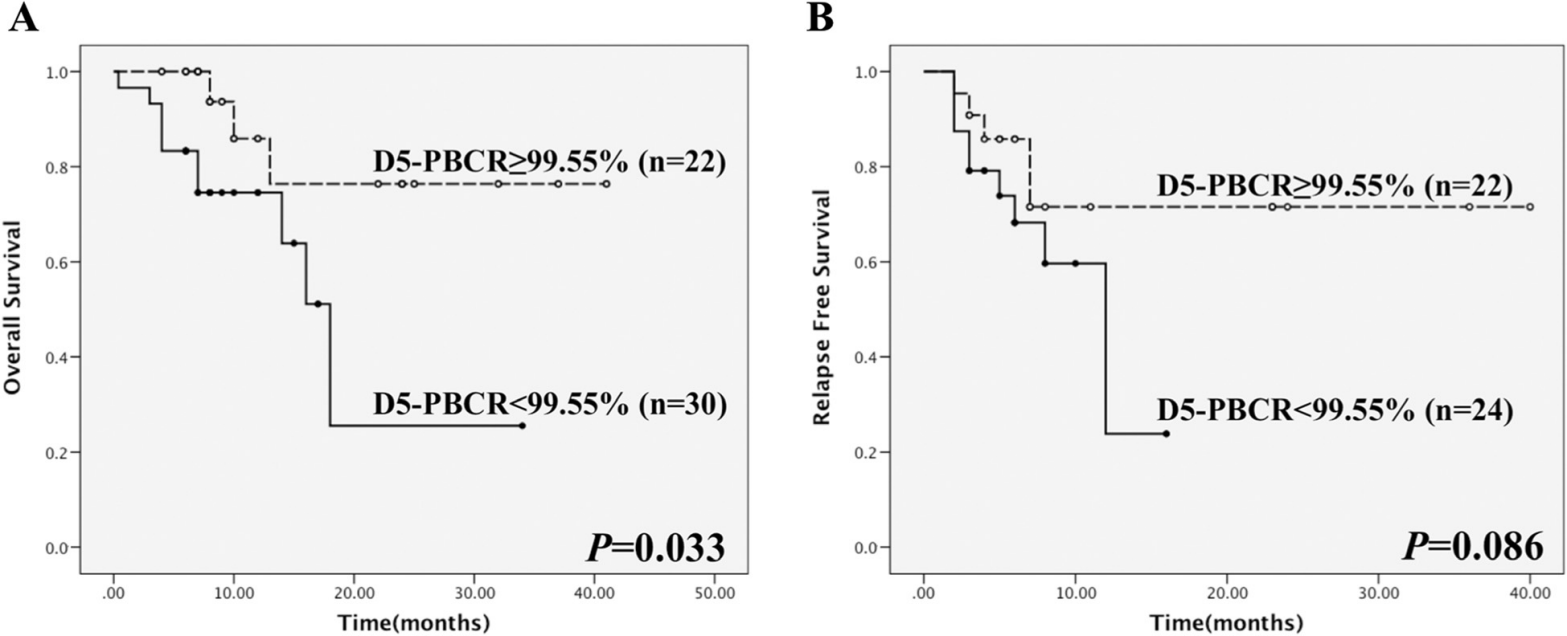
Survival analysis of patients in the cytogenetic-molecular intermediate-risk group. **(A)** OS of patients subdivided into high (≥99.55%) and low (<99.55%) D5-PBCR groups, *P* = 0.033. **(B)** RFS of patients subdivided into high (≥99.55%) and low (<99.55%) D5-PBCR groups, *P* = 0.086.

The distribution of the cytogenetic-molecular risk classification showed that more unfavorable cases were classified into the lower D5-PBCR (<99.55%) group (16/19, *P* = 0.019). In the intermediate risk group, although the distribution was similar, patients with low D5-PBCR showed significant adverse survival [estimated two-year overall survival (OS): 25.6% vs. 76.4%, *P* = 0.033; estimated two-year relapse-free survival (RFS): 23.9% vs. 71.5%, *P* = 0.086] (Figure 2A and B), even close to that of the unfavorable-risk group (Additional file 3: Figures S1A and S1B). In the favorable-risk group, no differences were observed in distribution or survival analysis.

**Figure 3:**
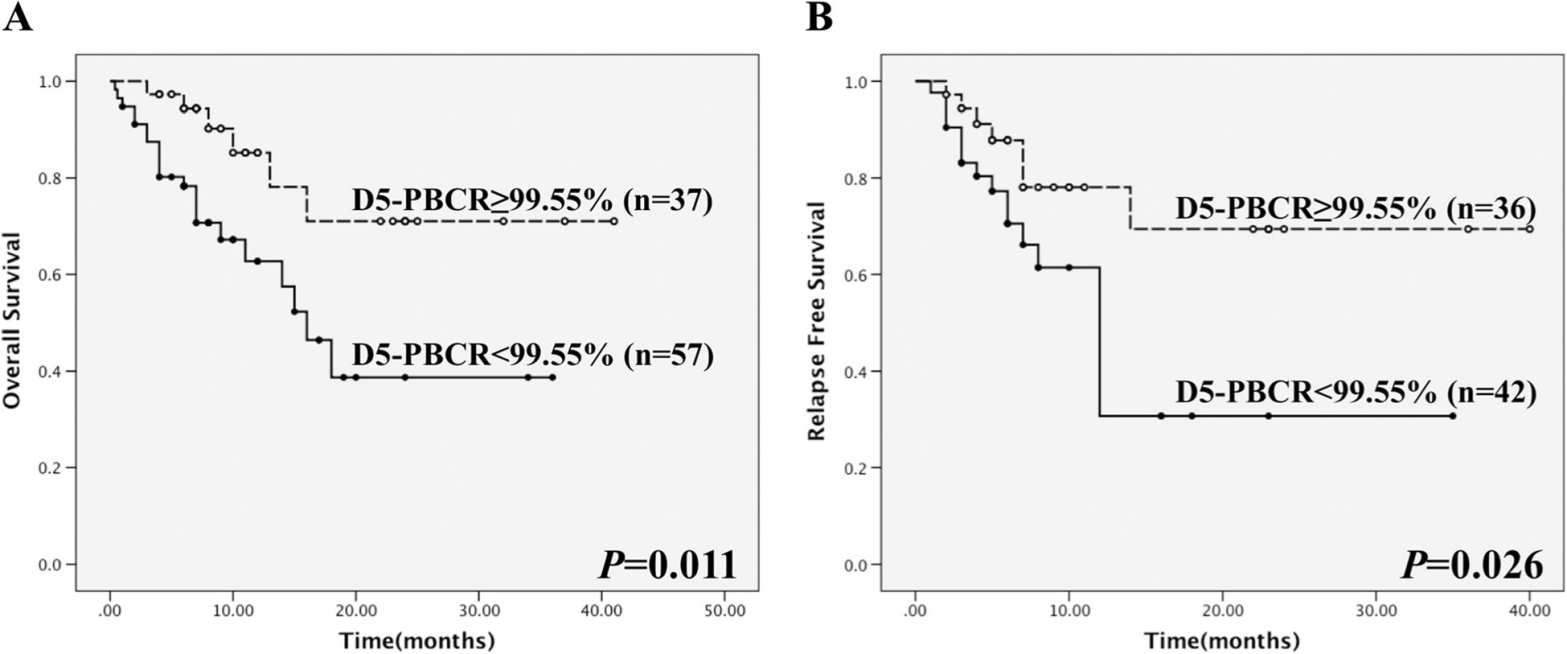
Survival analysis of whole cohort patients. **(A)** OS of patients subdivided into high (≥99.55%) and low (<99.55%) D5-PBCR groups, *P* = 0.011. **(B)** RFS of patients subdivided into high (≥99.55%) and low (<99.55%) D5-PBCR groups, *P* = 0.026.

Further analysis of the whole cohort showed that higher D5-PBCR (≥99.55%) was associated with significantly longer OS and RFS (estimated two-year OS: 71.0% vs. 38.7%, *P* = 0.011; estimated two-year RFS: 69.4% vs. 30.7%, *P* = 0.026; Figure 3A and B).

## Discussion

Based on clinical characteristics, cytogenetic and molecular markers, more precise prognostic stratification has been established in AML and diagnosis and treatment individualization has become feasible [3]. The establishment of an effective monitoring method is essential for the evaluation of the early response and the adjustment of treatment regimens and the improvement of the prognosis as well.

MRD detection by flow cytometry has been applied to identify subclinical levels of leukemia cells and evaluate treatment more precisely than conventional morphology; as such, this technique has been considered ideal for chemosensitivity assessment [6,17-21]. In ALL, MRD level (<0.01%) and cytogenetic-molecular markers are both considered as independent outcome predictors [8]. MRD-based clinical approaches in children and adult ALL have yielded excellent results, which confirmed that MRD can be effective in risk stratification and treatment intervention [22-25].

In AML, MRD monitoring also plays an important role in the evaluation of treatment effect. Retrospective studies have demonstrated a high prognostic value of post-induction MRD level in AML. Terwijn et al. [26] and Freeman et al. [27] defined 0.1% as the MRD cut-off value; in both studies, MRD is correlated with RFS but not with OS. However, Inaba et al. [28] reported that MRD has a limited value in childhood AML if measured by MFC. Thus far, the threshold of MRD related to prognosis of AML remains controversial. MRD cannot be considered as an independent prognostic predictor in AML, in contrast to ALL [26,27,29].

Either in ALL or AML, the time points of BM-MRD monitoring are not earlier than 2 weeks after the induction begins. Although prognostic correlation is excellent, the time at which correlation is determined may be too late for early intervention of induction regimen. Thus, studies have evaluated peripheral blast clearance in the first week of induction.

In patients with ALL, practical values of PB blast assessment have been reported. Gajjar et al. [10] found that the persistence of circulating blasts after 1 week of therapy is significantly related to the worse event-free survival in childhood ALL. Atsushi et al. [11] also presented the same conclusion in patients with ALL treated with prednisolone monotherapy. Studies related to AML have also shown that a rapid decrease in peripheral leukemic burden determined through either morphology or flow cytometry [12,15] is correlated with CR and long-term survival [14,16].

In this study, we monitored the early treatment response of 96 AML patients by detecting PB blasts through flow cytometry. The PBRR at all checkpoints (D_3_–D_9_) showed high prognostic value of CR (D_3_: *P* = 0.005, D_5_: *P* < 0.0001, D_7_: *P* < 0.0001, D_9_: *P* < 0.0001). Starting from D_5_, PBRR was significantly related to the total relapse rates (D_5_: *P* = 0.005, D_7_: *P* = 0.001, D_9_: *P* = 0.0005) and early relapse rates (D_5_: *P* = 0.017, D_7_: *P* = 0.008, D_9_: *P* = 0.009). This finding is consistent with that in published studies in which D_5_ is also determined as the median time of PB blast clearance [12-16]. The ROC analysis identified D5-PBCR of 99.55% initial blasts as the cut-off value. Patients with high D5-PBCR (≥99.55%) showed greater CR rates and less relapse rates (*P* < 0.001, *P* = 0.027) and were associated with significantly longer OS and RFS (*P* = 0.011, *P* = 0.026).

Our D5-PBCR cut-off value might help optimize current cytogenetic-molecular prognostic risk stratification. In our cohort, 84.21% (16/19) of unfavorable-risk patients were classified into low D5-PBCR group (<99.55%). In the intermediate-risk groups, D5-PBCR could further distinguish the subgroup of patients with relatively poorer prognosis, the two-year estimated OS and RFS rate were significantly worse in patients with D5-PBCR <99.55% (*P* = 0.033, *P* = 0.086), which were close to that of the unfavorable-risk group. This might facilitate further treatment regimen adjustments. The intermediate-risk patients with low D5-PBCR may be recommended for strengthening induction and consolidation therapy or receiving allo-HSCT.

D_5_ of induction is a valuable time point of early treatment response monitoring. This time point is appropriate to strengthen induction therapy. Augmented induction using dose-escalated regimens or three-drug combination has benefited patients [30-32]. Holowiecki et al. [31] also suggested that the addition of cladribine to the standard induction regimen can improve the outcome of patients with AML, particularly in the unfavorable-risk group. Thus, early risk evaluation by D5-PBCR provides the basis of individualized induction therapy.

The applicability of MRD by MFC is approximately 60% to 88% in patients with AML [6,17]. In patients with no LAIPs, BM MRD evaluation is unlikely performed through MFC assessment. In this case, D5-PBCR is considered as a new effective evaluation method. Almost all patients with AML suffer from circulating blasts at diagnosis, which enables PB blast monitoring a comparatively universal way. In our study, LAIP coverage was approximately 75% (72/96). Of the 24 patients with no LAIPs, 23 showed available data of D5-PBCR. The CR rate was 100% (7/7) in the high D5-PBCR group compared with 50% (8/16) in the low D5-PBCR group (*P* = 0.052); this result suggested that high D5-PBCR may help predict the CR status of patients without LAIPs.

However, for the few AML patients with very low percentage or even absent of PB blasts at diagnosis, BM MRD detection plays a more important role.

## Conclusions

An effective evaluation method of early treatment response was established by monitoring PB blasts through MFC. D5-PBCR cut-off value might help distinguish high-risk patients in the first week of induction; thus, prognostic predictive ability of current risk stratification can be improved and induction regimen modification can be performed.

To further establish practical and precise clinical guidance, more patients need to be accumulated and multicenter confirmation is required.

## Methods

### Patients and treatment protocols

A total of 96 patients with *de novo* AML (non-M3) were enrolled in this study from June 2011 to August 2014 in the Shanghai Institute of Hematology. The diagnosis and classification of the AML subtypes were established according to FAB [33] and WHO 2008 criteria [34]. The Ethics Committee of Ruijin hospital approved this study. All patients provided informed consent according to the Declaration of Helsinki.

All patients received standard first-line chemotherapy. The induction regimen consisted of idarubicin (8 to 10 mg/m^2^/d, D_1_–D_3_) or daunorubicin (45 to 60 mg/m^2^/d, D_1_–D_3_) and cytarabine (100 mg/m^2^/d, D_1_–D_7_). After CR was achieved, consolidation therapy was administered according to the patients’ cytogenetic-molecular risk stratification. Patients in favorable- and intermediate-risk group received HiDAC-based consolidation (2 g/m^2^, every 12 h, D_1_-D_3_), whereas patients in the unfavorable-risk group were recommended for allograft transplantation (*n* = 14) [30,35,36].

### Cytogenetic and molecular analysis

Cytogenetic data was available in 88 of 96 cases. The chromosomes were R-banded and/or G-banded in unstimulated BM cells after 24 h of culture. The karyotype was analyzed according to the International System for Human Cytogenetic Nomenclature (2009) [37].

Molecular studies were performed in 94 cases. Mutations of several genes, including *NPM1*, *NRAS*, *FLT3-ITD*, *FLT3-TKD*, *DNMT3A*, *CEBPA*, *MLL-PTD*, *C-KIT*, were detected through RT-PCR and sequencing. *MLL*-related fusion genes, namely, *MLL-AF9*, *MLL-AF10*, *MLL-AF6*, *MLL-ELL*, *MLL-ENL*, and *MLL-AF17*, were assessed through multiplex RT-PCR [36].

The cytogenetic-molecular risk groups were classified as follows: favorable group comprised t (8; 21), t (16; 16) or inv (16), and normal cytogenetics with *NPM1* mutation in the absence of *FLT3-ITD*, *DNMT3A*, and *MLL-PTD* mutations. Unfavorable group comprised 5/5q−,−7/7q−, inv (3)/t (3; 3), t (6; 9), complex karyotype (≥3 clonal chromosomal abnormalities), and normal cytogenetics with *FLT3-ITD*. The intermediate group consisted of other patients [35].

### Identification of LAIPs

Fresh BM or PB samples were processed according to the standard operating procedure of our institution [7]. The assays were performed using a 10-color flow cytometer (NAVIOS, Beckman Coulter, Brea, California, USA). Data were analyzed with KALUZA software (Beckman Coulter, Brea, California, USA). LAIPs were classified at diagnosis with different surface antigens (CD34, CD38, CD117, HLA-DR, CD13, CD33, CD14, CD15, CD64, CD2, CD4, CD7, CD11b, CD19, and CD56). The information on monoclonal antibody combination is shown in Additional file 4: Table S3.

More than 2.5 × 10^5^ events from PB samples and 1 × 10^6^ events from BM samples were required for flow cytometry detection to ensure sensitivity and accuracy. For BM samples, a cut-off value of 0.1% was determined as MRD positive.

### Calculation of PBRR and PBCR

PB samples of right before treatment (D_0_) and on the 3rd, 5th, 7th, 9th days (D_3, 5, 7, 9_) of the induction chemotherapy were collected. The absolute counts of blasts in PB were calculated using flow count fluorospheres (Beckman Coulter, Brea, California, USA). For patients with LAIPs, blasts with the specific immunophenotype were counted. For patients with no LAIPs, PB blasts were determined with surface antigens (including CD34+, CD117+, CD33+, CD13+, HLA-DR+, and CD45dim) associated with myeloid lineage cells. Patients whose PB blast percentage was higher than 0.5% at D_0_ were included in this study. PBRR was defined as the logarithmic ratio to the absolute counts of D_0_ [Log_10_ (D_0_/D_x_)] [38]. PBCR was defined as the percentage of absolute count reduction: [(D_0_ − D_x_)/ D_0_ × 100%].

### Statistical analysis

PBCRs were compared through independent-sample *t*-test. An ROC analysis was performed to evaluate the predictive efficiency of PBRR on patients’ CR. Statistical significance was considered when AUC was >0.6; the cut-off value was selected according to the maximum sum of sensitivity and specificity [39]. Chi-square test was performed to compare CR and relapse rates. OS was defined as the time of diagnosis to death or allo-HSCT or the last follow-up. RFS was defined as the time at which CR was achieved until relapse occurred and was also defined on the basis of whether a patient died or remained alive when CR was achieved at the last follow-up (censored). OS and RFS were estimated by using Kaplan-Meier method and compared by conducting log-rank test. The last follow-up was performed in December 2014, and the median follow-up time was 11 (0.4–41) months. Two-sided *P* < 0.05 was considered statistically significant. Statistical analyses were evaluated using SPSS 22.0 software (IBM, Armonk, NY, USA).

## Additional files

Additional file 1: Table S1. LAIP characteristics of patients. LAIPs were identified in 72 patients with four main types: 63 cases with cross-lineage antigen expression, 6 cases with asynchronous antigen expression, 13 cases with antigen dim/strong expression, and 8 cases with antigen expression missing. Additional file 2: Table S2. Gene mutation profile of cytogenetic-normal AML patients stratified by D5-PBCR. Mutations of several genes, including *NRAS*, *NPM1*, *FLT3-ITD*, *FLT3-TKD*, *DNMT3A*, *CEBPA*, *MLL-PTD*, *C-KIT*, and *MLL*-related fusion genes were detected in 58 cytogenetic-normal AML patients ,57 showed available data of D5-PBCR. A trend of more *NRAS* (14.8% vs. 0%, *P* = 0.051), *CEBPA* biallelic mutations (37.0% vs. 20.7%, *P* = 0.142), and less *MLL-PTD* mutations (3x.7% vs. 10.3%, *P* = 0.612) was observed in the D5-PBCR ≥ 99.55% group. Additional file 3: Figure S1. Survival analysis of patients with cytogenetic-molecular risk stratification. (A) OS of patients subdivided into favorable-risk group, intermediate-risk group with high D5-PBCR (≥99.55%), intermediate-risk group with low D5-PBCR (<99.55%), and unfavorable-risk group, *P* < 0.001. (B) RFS of patients subdivided into favorable-risk group, intermediate-risk group with high D5-PBCR (≥99.55%), intermediate-risk group with low D5-PBCR (<99.55%), and unfavorable-risk group, *P* = 0.010. Additional file 4: Table S3. The mAbs combinations utilized for MRD and PB blast assessment. The monoclonal antibody combination utilized for MRD and PB blast assessment included seven fixed mAbs (CD34, HLA-DR, CD13, CD33, CD117, CD10, and CD45) and eight alternative mAbs (CD2, CD4, CD7, CD19, CD56, CD11b, CD64, and CD14).

## Abbreviations

ALL: acute lymphoblastic leukemia
allo-HSCT: allogeneic hematopoietic cell transplantation
AUC: area under the curve
AML: acute myeloid leukemia
BM: bone marrow
CR: complete remission
FAB: French-American-British
LAIP: leukemia-associated aberrant immunophenotype
MRD: minimal residual disease
MFC: multiparameter flow cytometry
PB: peripheral blood
NCR: not achieved complete remission
OS: overall survival
PBCR: peripheral blasts clearance rate
PBRR: PB blasts reduction ratio
RFS: relapse-free survival
ROC: receiver operating characteristic
