# Supplementary material for: Clinical significance of day 5 peripheral blast clearance rate in the evaluation of early treatment response and prognosis of patients with acute myeloid leukemia
Source: J Hematol Oncol. 2015 May 10;8:48. doi: 10.1186/s13045-015-0145-1 (PMC4431040; doi:10.1186/s13045-015-0145-1)
Supplement: Additional file 1: Table S1. — LAIP characteristics of patients. LAIPs were identified in 72 patients with four main types: 63 cases with cross-lineage antigen expression, 6 cases with asynchronous antigen expression, 13 cases with antigen dim/strong expression, and 8 cases with antigen expression missing. [file 13045_2015_145_MOESM1_ESM.docx]

**Supplementary Table S1. LAIP characteristics of patients.**

| **LAIP** | | | **No.of cases** | |
| --- | --- | --- | --- | --- |
| ***Cross-lineage antigen expression***  **CD7+**CD34+ CD117+CD33+/dimCD13+/dimCD45dim  **CD2+**CD117dimCD34+CD33+CD13+CD45dim  **CD19+**CD117+CD34+CD13+/dimCD33+/dimCD45dim  **CD56+**CD117+/-CD34+/-CD33+/dimCD13+/dimCD45dim  **CD4**+CD117+CD34-CD33+CD13- HLA-DR+CD45dim  **CD56+CD19+**CD117+CD34+CD33+CD13+CD45dim  **CD7+CD56+**CD117+CD34+CD33+CD13+CD45dim  **CD4+CD56+**CD117+CD34+CD33+CD13+CD45dim | 30  1  7  16  1  3  4  1 | |  |  |
| ***Asynchronous antigen expression***  CD117+CD34+**CD11b+** CD33+CD13+CD45dim | 6 | |  |  |
| ***Antigen dim/strong expression***  CD19/56+CD117+CD34+**CD33dim** CD13+CD45dim  CD7/19/56+CD117+CD34+CD33+**CD13dim**HLA-DR+CD45dim  CD56+CD117+CD34+**CD33dimCD13dim**CD45dim | 8  3  2 | |  |  |
| ***Antigen expression missing***  CD19/56+CD34+CD117+CD33+CD13+**HLA-DR-**CD45dim  CD117-CD34-HLA-DR+CD33+**CD13-**CD45dim  CD117-CD34-HLA-DR+**CD33-**CD13+CD45dim | 3  2  3 | |  |  |
