# Supplementary material for: Clinical significance of day 5 peripheral blast clearance rate in the evaluation of early treatment response and prognosis of patients with acute myeloid leukemia
Source: J Hematol Oncol. 2015 May 10;8:48. doi: 10.1186/s13045-015-0145-1 (PMC4431040; doi:10.1186/s13045-015-0145-1)
Supplement: Additional file 2: Table S2. — Gene mutation profile of cytogenetic-normal AML patients stratified by D5-PBCR. Mutations of several genes, including NRAS, NPM1, FLT3-ITD, FLT3-TKD, DNMT3A, CEBPA, MLL-PTD, C-KIT, and MLL-related fusion genes were detected in 58 cytogenetic-normal AML patients ,57 showed available data of D5-PBCR. A trend of more NRAS (14.8% vs. 0%, P = 0.051), CEBPA biallelic mutations (37.0% vs. 20.7%, P = 0.142), and less MLL-PTD mutations (3x.7% vs. 10.3%, P = 0.612) was observed in the D5-PBCR ≥ 99.55% group. [file 13045_2015_145_MOESM2_ESM.docx]

**Supplementary Table S2.**

**Gene mutation profile of cytogenetic-normal AML patients stratified by D5-PBCR.**

| **Genes** | **D5-PBCR≥99.55%**  **(n=27)** | **D5-PBCR<99.55%**  **(n=30)** | ***P* value** |
| --- | --- | --- | --- |
| *NRAS* |  |  | 0.051 |
| Mutated | 4(14.8%) | 0(0%) |  |
| Wild type | 23(85.2%) | 28(100%) |  |
| Not available | 0 | 2 |  |
| *NPM1* |  |  | 0.469 |
| Mutated | 7(25.9%) | 5(17.9%) |  |
| Wild type | 20(74.1%) | 23(82.1%) |  |
| Not available | 0 | 2 |  |
| *FLT3-ITD* |  |  | 1.000 |
| Mutated | 3(11.1%) | 3(10.7%) |  |
| Wild type | 24(88.9%) | 25(89.3%) |  |
| Not available | 0 | 2 |  |
| *FLT3-TKD* |  |  | 1.000 |
| Mutated | 0(0%) | 1(3.4%) |  |
| Wild type | 27(100%) | 28(96.6%) |  |
| Not available | 0 | 1 |  |
| *DNMT3A* |  |  | 0.837 |
| Mutated | 6(22.2%) | 6(20.0%) |  |
| Wild type | 21(77.8%) | 24(80.0%) |  |
| Not available | 0 | 0 |  |
| *CEBPA* |  |  |  |
| Biallelic | 10(37.0%) | 6(20.7%) | 0.142 |
| Single | 2(7.4%) | 1(3.4%) | 0.565 |
| Wild type | 15(55.6%) | 22(75.9%) |  |
| Not available | 0 | 1 |  |
| *MLL-PTD* |  |  | 0.612 |
| Positive | 1(3.7%) | 3(10.3%) |  |
| Negative | 26(96.3%) | 26(89.7%) |  |
| Not available | 0 | 1 |  |
| *MLL fusion* |  |  | 1.000 |
| Positive | 1(3.7%) | 1(3.4%) |  |
| Negative | 26(96.3%) | 28(96.6%) |  |
| Not available | 0 | 1 |  |
| *CKIT* |  |  | NA |
| Mutated | 0(0%) | 0(0%) |  |
| Wild type | 26(100%) | 29(100%) |  |
| Not available | 1 | 1 |  |
