# Supplementary figures and images for: Clinical significance of day 5 peripheral blast clearance rate in the evaluation of early treatment response and prognosis of patients with acute myeloid leukemia
Source: J Hematol Oncol. 2015 May 10;8:48. doi: 10.1186/s13045-015-0145-1 (PMC4431040; doi:10.1186/s13045-015-0145-1)

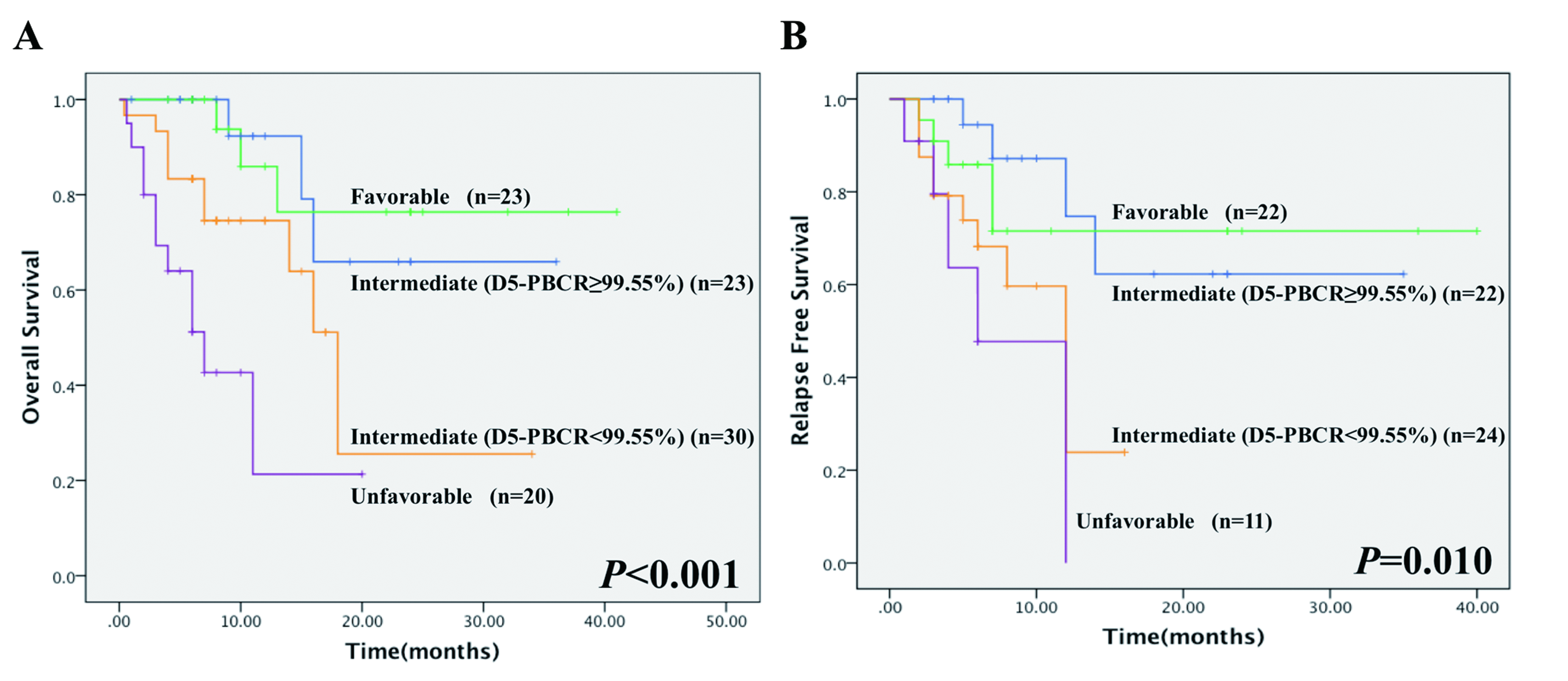

Supplement: Additional file 3: Figure S1. — Survival analysis of patients with cytogenetic-molecular risk stratification. (A) OS of patients subdivided into favorable-risk group, intermediate-risk group with high D5-PBCR (≥99.55%), intermediate-risk group with low D5-PBCR (<99.55%), and unfavorable-risk group, P < 0.001. (B) RFS of patients subdivided into favorable-risk group, intermediate-risk group with high D5-PBCR (≥99.55%), intermediate-risk group with low D5-PBCR (<99.55%), and unfavorable-risk group, P = 0.010. [file 13045_2015_145_MOESM3_ESM.tif]
