# Supplementary material for: Clinical significance of day 5 peripheral blast clearance rate in the evaluation of early treatment response and prognosis of patients with acute myeloid leukemia
Source: J Hematol Oncol. 2015 May 10;8:48. doi: 10.1186/s13045-015-0145-1 (PMC4431040; doi:10.1186/s13045-015-0145-1)
Supplement: Additional file 4: Table S3. — The mAbs combinations utilized for MRD and PB blast assessment. The monoclonal antibody combination utilized for MRD and PB blast assessment included seven fixed mAbs (CD34, HLA-DR, CD13, CD33, CD117, CD10, and CD45) and eight alternative mAbs (CD2, CD4, CD7, CD19, CD56, CD11b, CD64, and CD14). [file 13045_2015_145_MOESM4_ESM.docx]

**Supplementary Table S3.**

**The mAbs combinations utilized for MRD and PB blast assessment.**

| **Seven fixed mAbs** | **Alternative mAbs** |
| --- | --- |
| CD34-ECD | CD2-PE |
| HLA-DR- APC-CY7 | CD4-PE |
| CD13-APC | CD7-PE |
| CD33- PE-Cy5 | CD19-PE |
| CD117-PE-Cy7 | CD56-PE |
| CD10-PB | CD11b-FITC |
| CD45-PO | CD64-FITC |
|  | CD14-A700 |

Abbreviations: mAbs, monoclonal antibodies; A700, alexafluor 700; APC, allophycocyanin; APC-CY7, allophycocyanin-Cy7; ECD, energy coupled dye; FITC,fluoresceinisothiocyanate; PB, pacific blue; PC5, phycoerythrin-Cy5; PC7, phycoerythrin-Cy7;PE, phycoerythrin; Po, pacific orange.
